# Editorial: Membrane Structure and Dynamics Studied With Neutron Scattering

**DOI:** 10.3389/fchem.2021.732062

**Published:** 2021-08-19

**Authors:** O. Holderer, A. Koutsioubas, C. J. Garvey, T. Nylander

**Affiliations:** ^1^ Jülich Centre for Neutron Science (JCNS) at Heinz Maier-Leibnitz Zentrum (MLZ), Forschungszentrum Jülich GmbH, Garching, Germany; ^2^ Lund Institute for Advanced Neutron and X-ray Science, Lund, Sweden; ^3^ Heinz Maier-Leibnitz Zentrum (MLZ), Technische Universität München, Garching, Germany; ^4^ Department of Chemistry, Lund University, Lund, Sweden

**Keywords:** microemulsions, lipid membranes, bending elasticity, neutron scattering, neutron spin echo, reflectometry, small angle neutron scattering

Self-assembled phospholipid bilayers are ubiquitous in biology and chemistry. In biological systems they constitute membranes that act as cell barriers that mediate the exchange of compounds, energy and information with the extra-cellular environement and essentially act as a host for membrane proteins that carry out a multitude of critical taks. In modern chemistry, self-assembled surfactant or lipid membranes in dispersed lamellar or non-lamellar phases (cubic and hexagonal) or bicontinuous microemulsions are frequently used in health care or consumer products to encapsulate and deliver active substances for controlled pharmacokinetics. Thanks to their generally well defined structure and properties, and using nature as an inspiration they can also be used to formulate nano-scale reactors for sustainable synthesis.

**FIGURE 1:**
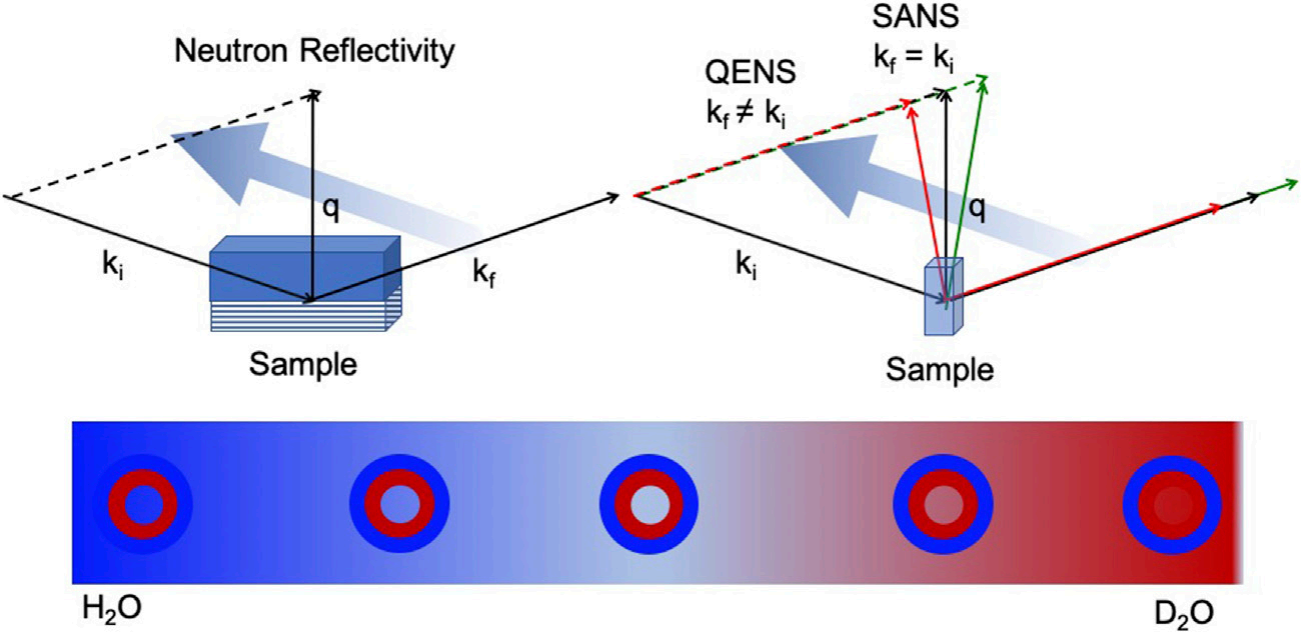
Self assembled membranes are ubiquious in chemical systems, such as surfactant membranes, and biological systems as for example phospholipid membranes. Neutron reflectivity **(left)** and scattering **(right)** allows to study the structure and motion in a realistic environment, e.g., physiological conditions. By isotop substitution it is possible to highlight different parts of the sample under chemically quasi identical conditions **(bottom)**.

Neutron scattering provides a unique view on nanoscopic and mesoscopic structure of self-assembled membranes, for example with Small Angle Neutron Scattering (SANS) in solution or with Neutron Reflectivity (NR) using contrast variation through deuterium labelling (Figure 1). Thermal membrane fluctuations are measured with Neutron Spin Echo (NSE) spectroscopy, allowing us to study how changes in environment, for example adding diblock copolymers or changing solvent properties, can influence membrane elasticity. Recent advances in instrumentation have also provided access to membrane dynamics at the solid-liquid interface with grazing incidence NSE (GINSES).

Contrast variation by deuteration in neutron scattering provides a tool to get insight into the properties of individual components in the liquid state on nanometer length scales that is not accessible through other techniques.

The collection of papers in this issue only covers a fraction of the plethora of membrane properties that can be studied with state-of-the-art neutron scattering techniques.

The interaction of ionic liquids (ILs) with cellular membranes is studied in the Article of Sharma et al. ILs as solvents with increasing importance in chemical engineering processes of various kinds are generally toxic to living organisms. The article aims therefore in a better understanding of the interaction if ILs with lipid membranes by studying the influence of ILs on the membrane fluidity and molecular motion and learning in this way how to work with or use safely ILs. Quasi elastic neutron scattering was the method of choice to get insight into the molecular dynamics at the membrane-IL-interface.

A completely different environment is studied in the contribution of LoRicco et al. with neutron diffraction. Apolar molecules stabilize the membrane of archea for survival in the most extreme conditions of high temperatures and pressures.

The interaction of lipase with microemulsion membranes has been investigated by Engelskirchen et al. in view of understanding and improving lipase catalyzed reactions. Combining structural and spectroscopic investigations were essential for getting hints on the residence time of the lipase at the surfactant membrane.

The strength of neutron scattering in soft matter and biology is the accessibility to parts of a complex sample through contrast variation. This has been used by Conn et al. to study membrane proteins, here in the bicontinuous cubic phase of phospholipid bilayers.

Thermally driven motion of membranes or incorporated proteins can be investigated with neutron spin echo (NSE) spectroscopy on nanometer and nanosecond length- and time-scales under physiologically relevant conditions. Subtleties of modern NSE experiments are discussed in the Article of Hoffmann.

An example of studies of the membrane dynamics with NSE is found in the contribution of Jaksch et al., where, in combination with reflectometry, the influence of salt on phospholipid membranes is studied.

Also plant cells contain membranes, and photosynthetic membranes are eminently important for nature and thus studied since long time. Combining different techniques such as microscopy and x-ray or neutron scattering provides an added value helping to understand nature, when the experimental results are properly modelled, as is shown in the paper of Jakubauskas et al..

Use of (partial) deuteration is a strong argument for neutron scattering techniques, which is applied for studying the interplay between high density lipoproteins (HDLs), cholesterol and the lipid membrane to help understanding the factors for deseases as Altzheimer’s or atherosclerosis in the article by Waldie et al.).

Neutron diffraction on stacks of lipid multilayers allowed Luchini et al. to characterize different preparation methods, an important prerequisite for reliable data analysis, and further on the existence of different lipid phases could be proven by the diffraction data attributed to the heterogeneity of their acyl chain composition. Using natural extracts with the ability of producing deuterated lipid mixtures plays again with the power of adjustable contrast for biological samples.

Neutron reflectivity is mainly used in the contribution by Krugmann et al. in order to investigate the nanoscopic details of the role of myelin basic protein in the formation of the sheath that wraps around axons. Combination of static and kinetic measurements together with theoretical arguments provide insights concerning the myelination process.

Finally the review article by Kinnun et al. highlights the advantages of contrast variation availiable in various neutron methods for accessing structural and dynamic information about the in-plane and out-of-plane structure of a variety of biomembrane systems.

With this research topic we hope to provide a useful and interesting overview over some recent advances in membrane studies with different neutron scattering techniques and to illustrate the potential of the different techniques of diffraction, reflectivity and spectroscopy measurements which allow together with the unique contrast variation possibilities with isotope substitution a nanoscopic view into the details of artificial and natural self assembled membranes.

